# New VSED Advance Directive: Improved Documentation to Avoid Late-Stage Dementia

**DOI:** 10.1017/jme.2025.10176

**Published:** 2025

**Authors:** Thaddeus Mason Pope, Lisa E. Brodoff, Erin Mae Glass, Paul T. Menzel, Robb M. Miller

**Affiliations:** 1 Mitchell Hamline School of Law, United States; 2 Seattle University, United States; 3 Chuckanut Law, United States; 4 Pacific Lutheran University, United States; 5 Independent, United States

**Keywords:** VSED, Dementia, Advance Directive, Dementia Directive, End of Life, Minimal Comfort Feeding, MCF

## Abstract

People use advance directives to express preferences that direct their future care when they lack decision-making capacity. One form of advance directive, a “dementia directive,” records preferences about living in various stages of dementia. This is important because many Americans want to avoid living with advanced progressive dementia. Unfortunately, traditional advance directives cannot dependably achieve this goal. In contrast, some dementia directives can achieve this goal, by directing cessation of manually assisted feeding and drinking.

While many dementia directives have been published, most have gaps and omissions that thwart the goal of avoiding extended intolerable life in advanced dementia. To overcome these problems, we formulated a new dementia directive. This article explains the value of this new directive. We proceed in six stages. First, we review the prevalence of advanced dementia. Second, we identify the disadvantages of another option for accomplishing the goal of not living into advanced dementia, preemptive VSED. Third, we distinguish notable court cases where dementia directives were unsuccessful. Fourth, we review nine prominent dementia directives, noting how the Northwest Justice Project’s Advance Directive for VSED remedies those shortcomings. Fifth, we review this directive’s legal status. Sixth, we articulate its ethical justification.

## Introduction

People use advance directives to express preferences that direct their future healthcare when they lack decision-making capacity.^1^ A particular form of advance directive, a “dementia directive,” records a person’s attitudes and preferences about living in various stages of dementia.^2^ This is important because many Americans want to avoid living with advanced progressive dementia.^3^

Unfortunately, traditional advance directives cannot dependably achieve this goal, because people with dementia can live many years without reaching the point when most advance directives would direct the withholding of life-sustaining treatment.^4^ Dementia directives can fill this gap. If they include instructions to cease manually assisted feeding and drinking, dementia directives offer patients a way to accomplish their goal of ending life when they regard its continuation as intolerable.

We acknowledge the growth in advance directives for euthanasia.^5^ Many, such as those newly authorized in Quebec, are primarily for persons who are determined to avoid living into late-stage dementia.^6^ But these directives are not available to US citizens, or those in many other countries. All US jurisdictions that authorize medical aid in dying exclude the possibility of advance requests because they require that the individual: (1) make a contemporaneous request with both (2) decision-making capacity and (3) a terminal illness.^7^ Accordingly, we exclude euthanasia directives and focus on only those dementia directives now authorized in the United States.^8^

For persons with decision-making capacity, voluntarily stopping eating and drinking (VSED) is a legally permissible option for hastening death that patients already regularly use to avoid living in late-stage dementia. With proper palliative support, it is typically a relatively peaceful and comfortable death.^9^ Of course, this evidence is based on VSED cases where patients were supported with expert palliative care. Dementia directives combine this right to VSED with the established right of people to control their lives after they lose capacity.^10^ These advance directives are especially relevant to those who want to avoid living with severe dementia.

Many “dementia directives” have been posted and published.^11^ But only those that direct withholding oral food and drink are likely to be useful in avoiding extended life in advanced dementia.^12^ Unfortunately, most posted and published dementia directives have gaps and omissions that thwart the goal of avoiding extended life in advanced dementia.^13^

To overcome these problems and better meet the ethical, legal, clinical, institutional, and social challenges faced by advance directives for VSED, we formed the National VSED Advance Directive Committee.^14^ We then formulated a new dementia directive: the Northwest Justice Project VSED Directive (or NJP directive). It is posted online as a fillable form and freely available on the NJP website and as an Appendix to this article.^15^

The Northwest Justice Project is a nonprofit legal aid program that delivers free legal services and extensive self-help and educational materials to low-income and elder clients throughout Washington State.^16^ One of the authors, Professor Lisa Brodoff, has worked with NJP attorneys for the last two decades to create easily accessible online self-help advanced directives and powers of attorney for anyone to use to do their own healthcare and advanced care planning.

Over the last five years, the NJP attorneys along with Professor Brodoff moved all of the advanced directives and powers of attorney created into an auto-fill format where users answer questions and receive information that leads to a final legal document generated for printout and execution.^17^ This online resource has been extremely successful, and the use of advanced directives has skyrocketed especially since the creation of the auto-fill format. As a result, when the National VSED Advance Directive Committee created this new VSED directive, it was a natural fit to use NJP’s easily accessible website and auto-fill format to make it readily available on a nationwide basis.

This article explains the value of this new dementia directive. We proceed in six stages. First, we review the prevalence of advanced dementia. Second, we identify the disadvantages of preemptive VSED. Third, we describe two notable court cases where dementia directives were unsuccessful. Fourth, we review nine prominent dementia directives, noting how the NJP directive remedies their shortcomings. Fifth, we review the NJP directive’s legal status. Sixth, we articulate its ethical justification.

## Prevalence of Advanced Dementia

Progressive dementia, most commonly in the form of Alzheimer’s disease, afflicts more than six million Americans. Experts estimate that, by 2060, 13 million will be afflicted, including 6 million over the age of 85.^18^ Although the percentage in the advanced, severe stages is unknown, experts estimate that hundreds of thousands of Americans die from dementia, as distinct from dying merely with dementia.^19^

Since people die from dementia only in its most advanced stages, a reasonable, though rough, estimate from this data is that more than several hundred thousand in the US currently suffer from severe dementia. Because advanced dementia lasts for years and because this prevalence is high, it is important to examine how, legally and ethically, people may effectively avoid this fate, if that is their wish.

We acknowledge that substantial evidence shows that stigma negatively impacts the lives of people living with dementia, their families, and their caregivers. Many individuals make harmful and misleading assumptions about dementia.^20^ But at the latest stages of dementia, there are some hard truths. These patients cannot engage either in instrumental activities of daily living (such as using public transportation, managing finances, or shopping) or even in basic activities of daily living (such as bathing, getting dressed, or eating).^21^ So patients may not be driven by bias and stereotypes. They may accurately perceive and fear cognitive loss, dependence on others for their basic needs, and suffering for both themselves and their loved ones.^22^ Moreover, many of those who go to the length of writing a relatively unusual advance directive, one for VSED, are led to do so in significant part because of their experience with those who have lived into advanced dementia, often for years. The realities of such life, not social stigma, explain their decision.

## VSED: Giving Up Good Days to Avoid Bad Years

Some individuals want to avoid living into advanced dementia so strongly that they are willing to sacrifice some good time yet remaining in their lives by hastening their deaths now. Some resort to medical aid in dying in Switzerland, to non-medical end-of-life options, or even to suicide while they still have decision-making capacity.^23^ Others avoid living into advanced dementia by “preemptive VSED.”^24^ They made a deliberate choice to stop eating and drinking. Once these patients start VSED and cease the intake of any amount of food or water by mouth, they will die in about 8 to 14 days.^25^ VSED is widely recognized as a compassionate option for hastening death. And it is supported by clinical practice guidelines.^26^ With significant caregiving and palliative care support, VSED deaths are mostly reported to be peaceful and comfortable.^27^

These patients are motivated in part by the risk they see that any directive for VSED they write will not be honored. Such noncompliance would condemn them to the fate they are so determined to avoid. We present two such cases that graphically illustrate the patient determination required, and the pressure created by a closing window of capacity.

### Jane’s Preemptive VSED

Jane loved life deeply. She was a voracious reader. She loved her family, gardening, and adventures in the mountains with her beloved husband. Jane had a history of Alzheimer’s in her family. Her grandmother, mother, and brother all suffered from the disease. When Jane was diagnosed with mild cognitive impairment in 2010, she knew it was likely the first step towards her own path with Alzheimer’s. She committed to living a full and adventurous life as long as possible.

But when her impairment turned into an official dementia diagnosis and her quality of life began to diminish, Jane researched her end-of-life options. She did not want to live with the late stages of her diagnosis. Jane chose to hasten her death using VSED while she still had decisional capacity, knowing that she would be giving up some acceptable quality of life ahead. It brought her great peace to have that autonomy. Jane began VSED on February 1, 2020, and died surrounded by loving family and friends at home on February 10, 2020.^28^

### Cindy’s Preemptive VSED

Cindy was a palliative medicine physician and had treated patients with Parkinson’s, liver failure, dementia, and other incurable conditions with long and unpredictable trajectories. Cindy’s mother and her father had both died in severe dementia, with long and difficult last years of falls, agitation, hallucinations, and the blank stare of nonengagement. Once diagnosed with her own early-onset Alzheimer’s, Cindy explained in a video statement: “I choose to die by VSED…. I don’t want the long, relentless decline and complete loss of self that mark the late stages…. My choice is to give up a few good days to avoid bad years…. It may seem too early for many, but I don’t want to miss my window of opportunity.” Cindy began VSED on March 28, 2022, and died at home on April 13, 2022.^29^

### Lessons from Jane and Cindy

Both Jane and Cindy used preemptive VSED to allow their deaths to occur while they still had decision-making capacity. They both made this deliberate choice to give up good months (and possibly years) to ensure that they avoided late-stage dementia and the possibility that their end-of-life wishes would not be honored. But is this sacrifice necessary? Is the “now or never” dilemma unavoidable?

There should be ways for Jane and Cindy to accomplish their goals without sacrificing life that they would still like to live. It is one thing for a patient to give up life under circumstances they find intolerable. For fifty years, law and policy have regarded this as a right.^30^ But it is quite another thing for a patient to give up life that they still find worthwhile. That should be avoided.

The use of preemptive VSED is driven not only by the strong desire to avoid living into severe dementia, but also typically by the risk and fear that a VSED directive will not later be honored by medical providers and caregivers. If a VSED directive could be written, and the relevant parties communicated with, sufficiently well to create confidence that it will be followed, people would be less inclined — like Jane and Cindy were — to preemptively stop eating and drinking while they still have an acceptable quality of life. Can a VSED directive be created that would reasonably provide this confidence and peace of mind? That was our goal in developing the NJP advance directive.

## Unsuccessful Dementia Directives — Nora Harris and Margot Bentley

Jane and Cindy’s fear that a VSED directive would not be honored is illustrated by two well-publicized cases that graphically and movingly illustrate what can go wrong with an inadequate advance directive to withhold oral food and drink. We mitigate these inadequacies with the new NJP directive.

### Nora Harris (Oregon)

In 2017 in Medford, Oregon, Nora Harris, 64, died at a long-term care center eight years after being diagnosed with early-onset Alzheimer’s. In her 2009 advance directive, Harris called for no measures to prolong her life and explicitly granted her husband, as her healthcare agent, the power to refuse “artificial nutrition and hydration (nourishment provided by feeding tube) (commencement or termination).” But the directive did not specifically address oral feeding and drinking.^31^

Later, consistent with Harris’ stated wishes, Harris’ husband asked that the care facility stop spoon-feeding. The care facility refused, and the husband went to court to enforce his wife’s wishes. The court denied his request, citing state law requiring the center to offer residents adequate meals, including manual assistance with feeding.^32^ So, spoon-feeding continued in Harris’ last months, even after she became bedridden. Finally, her kidneys failed, and she died.^33^

### Margot Bentley (British Columbia)

Margot Bentley was 85 when she died in November 2016, in Abbotsford, British Columbia, 17 years after her Alzheimer’s diagnosis. In her work as a nurse, Bentley often saw people lingering with Alzheimer’s and regularly said that she “didn’t want that happening to her.” She had frequent conversations with her children to the effect that “non-responsive” people should be allowed to die. Accordingly, in her 1991 directive, Bentley requested that she receive “no nourishment or liquids” if she developed an incurable illness. But, like Harris, Bentley did not specifically address oral food and drink.^34^

During the five years before her death, Bentley’s daughters pleaded in vain with the nursing home and the courts to respect their mother’s wishes and not prolong her life with persistent spoon feeding. Ultimately Bentley succumbed to the exact fate she had worked so hard to avoid. She remained in a non-responsive state for years, kept alive by the nursing home’s spoon-feeding.

### Lessons from Harris and Bentley

Various factors prevented family members from having manually assisted oral food and drink withheld from Harris and Bentley. Key among them were insufficient advance directives. Both Harris and Bentley failed to make explicit that they wanted caretakers to withhold manually assisted oral food and drink, not just artificial feeding tubes and IVs. While Bentley requested that she receive “no nourishment or liquids” if she developed an incurable illness, she did not specifically reference “oral” food and drink, “spoon” feeding, or “hand” feeding. Nor did she identify which symptoms of advanced dementia should trigger withholding. Bentley also failed to specify what should be done if she continued to accept oral food and drink by opening her mouth and swallowing.

To be effective in accomplishing the goal of not living into advanced dementia, a dementia directive must clearly address five critical questions:

1. What is to be withheld — specifically, does that include manually assisted oral food and drink?
2. When should it be withheld — what symptoms need to be reached for food and drink to be withheld?
3. What degree of palliative measures should be employed when they are withheld?
4. What should be done if the directive will not be implemented?
5. What should be done if, when food and drink are withheld, the person seems to express a desire to eat or drink?

## The NJP Directive’s Advantages over Other Dementia Directives

Nine notable other published dementia directives have been published by: (1) Caring Advocates, (2) dementia-directive.org (associated with Barack Gaster), (3) Compassion and Choices, (4) End of Life Washington, (5) End of Life Choices New York, (6) Dartmouth College, (7) Life Circle, (8) Final Exodus, and (9) the Final Exit Network.^35^

These directives are incomplete.^36^ Some offer detailed descriptions of the stages of dementia and pose multiple choices for each. Others are relatively simple and much less detailed. Most explicitly speak to withholding manually assisted oral food and drink. But compared to the new NJP directive, they have serious shortcomings.^37^

In summary, categorized by the five previously stated critical questions, key shortcomings of the nine most notable dementia directives include:

What is to be withheld?

- Failing to address at all the possibility of withholding oral food and drink (Gaster).

When is it to be withheld?

- Failing to focus on more than levels of “suffering” in eliciting the person’s preferences for when to withhold oral food and drink – a deficiency when the person in advanced dementia is apparently not suffering, but passively “content” (Caring Advocates, End of Life Choices NY).^38^
- Failing to indicate whether withholding should still begin if, though the triggering conditions have been met, the person seems comfortable or happy and one’s designated agent believes quality of life is still sufficient (Gaster, End of Life WA, End of Life Choices NY, Dartmouth, Life Circle, Final Exit Network).
- Failing to handle the situation where, at the designated time for implementation, the person appears to want to be fed, e.g., by opening their mouth reflexively and swallowing (Gaster).

What degree of palliative measures should be employed?

- Failing to indicate the extent of palliative care to use when food and drink are withheld, including intensive measures such as palliative sedation (Gaster, Compassion & Choices, End of Life WA, End of Life Choices NY, Dartmouth, Final Exodus, Final Exit Network). Good palliative support is typically crucial for a death by withholding food and drink to be peaceful and comfortable.

What to do if the directive will not be implemented?

- Failure to indicate what to do if medical facilities or providers will not honor the directive, including refusals based on conscience (Gaster, Compassion and Choices, End of Life Choices NY, Dartmouth, Life Circle, Final Exodus, Final Exit Network).
- Failing to provide an option for “minimal comfort feeding” in situations where for any variety of reasons the directive to withhold all assisted oral food and drink will not be implemented (Caring Advocates, Gaster, Compassion & Choices, End of Life WA, End of Life Choices NY, Dartmouth, Life Circle, Final Exodus, Final Exit Network).^39^

What to do if a desire to eat or drink is expressed after food or drink is withheld?

- Failing to address the situation where the person appears distressed when food or drink are withheld (Gaster, End of Life Choices NY, Dartmouth, Life Circle, Final Exodus).
- Failing to provide an option for shifting from withholding all food and drink to “minimal comfort feeding” if the person expresses a desire to eat or drink when food and drink are withheld (Caring Advocates, Gaster, Compassion & Choices, End of Life WA, End of Life Choices NY, Life Circle, Final Exodus, Final Exit Network).^40^

As controversial as dementia directives can be, a directive must speak to these situations if it is to provide any realistic, reasonable assurance that it will be honored. Legal and ethical uncertainty will plague directives that do not. While other directives often address many of these situations, the Northwest Justice Project (NJP) directive speaks to them all. We hope that as understanding of what is necessary improves, other directives will also include all these essential elements.

Indeed, End of Life Washington has recently removed its directive and instead offers the NJP directive.^41^ Final Exit Network now also offers the NJP directive, explaining it as “Our Cover-Your-Bases Favorite — It’s More Complicated, but Addresses More Potential Issues.”^42^ Again, Table 1 presents the directives and the elements they do or do not address.

**Table 1.** Comparison of Leading Dementia Directives

	Addresses manually assisted oral food and water	Addresses more than suffering on when to withhold food and water	Addresses when legal decision maker believes quality of life is still sufficient	Addresses when person appears to want food and water	Addresses extent of palliative care, including palliative sedation	Addresses when facility or provider will not honor directive	Addresses option for minimal comfort feeding if VSED not possible	Addresses when person appears distressed by food and water being withheld	Addresses option for minimal comfort feeding if person expresses persistent desire to eat and drink
Northwest Justice Project^23^	√	√	√	√	√	√	√	√	√
Caring Advocates (Terman)^21^	√		√	√	√	√		√	
Compassion and Choices^20^	√	√	√^*^	√				√	
Dartmouth^16^	√	√		√					√^**^
dementia-directive.org (Gaster)^13^^–^^14^		√							
End of Life Choices New York^17^	√			√					
End of Life Washington^18^	√	√		√		√		√	
Final Exit Network^19^	√	√		√				√	
Final Exodus^22^	√	√	√	√					
Life Circle^15^	√	√		√	√				

* Addresses only what to do when the supervising clinician, not the patient’s agent, believes quality of life is still sufficient.

** Offers “comfort feeding only,” not minimal comfort feeding, if person expresses persistent desire to eat and drink.

## Legal Status of VSED Directives

An individual’s right to direct withholding or withdrawing life-sustaining interventions is firmly grounded in constitutional, statutory, and common law.^43^ This right includes the right to direct future care decisions, including the right to refuse future manual feeding and drinking. Every state has statutes that govern advanced directives.^44^

Admittedly, language in statutory advanced directive forms varies substantially from state to state.^45^ And most state statutes do not directly address VSED or decisions involving future personal care preferences such as manual administration of food and drink. But, because the right to direct withholding or withdrawing life-sustaining interventions is constitutionally protected and well-established, the right to direct withholding of manually assisted food and drink by advance directive should apply in each state even if the state advance directive statute does not specifically mention VSED.^46^

In some state statutes, VSED falls squarely within the scope of instructions permitted in an advance directive. Nevada, for example, includes an “End-of-Life Decisions Addendum Statement of Desires” designed for “adults with dementia.”^47^ This addendum explicitly prompts the individual to answer whether they “want to get food and water even if I do not want to take medicine or receive treatment.”^48^

Similarly, Vermont permits advance directives to address not only “health care” but also “personal circumstances.”^49^ Furthermore, Vermont clarifies that advance directives may address “services to assist in activities of daily living,” which includes assisted feeding.^50^ Likewise, other states are also considering legislation that would clarify that advance directives may address “how and under what circumstances the ingestion of food and liquids may be limited or discontinued.”^51^

Most states are not as direct and explicit as Nevada and Vermont. Most simply permit individuals to record instructions regarding their future “health care,” which is defined as “any care, treatment, service, or procedure to maintain, diagnose, or otherwise affect a person’s physical or mental condition.”^52^ Still, even without directly and explicitly including VSED, the broad scope of this definition should include the right to direct whether one wants to receive manually assisted food and drink. Indeed, the 2023 Uniform Health Care Decisions Act (UHCDA) advises that “health care” should “be given the broadest possible construction” and includes “personal and custodial care.”^53^ In 2025, both Delaware and Utah adopted the UHCDA.^54^ And several more states considered adoption.^55^

Furthermore, many state statutes authorize individuals to supplement the state statutory form language to express additional decisions relevant to future care decisions.^56^ And state statutes grant legal immunity for good faith compliance with a patient’s advanced directive.^57^

Unfortunately, some medical providers still adhere only to their state’s specific statutory directive language and disregard additional patient decisions rejecting care, though those patient decisions are constitutionally, and often, in fact, statutorily protected.

A different and more difficult legal question arises when someone with a VSED directive makes a gesture or utterance indicating a desire for food or drink after they have lost decisional capacity. In most states, any such gesture or utterance lacks legal significance because the patient who makes such an utterance lacks decisional capacity at that time.^58^ So the advance directive directing VSED remains valid and in force.^59^ Patients can achieve the same result even in states that do not require capacity to revoke or modify an advance directive. Patients can specifically include a provision (known as a Ulysses clause) directing caregivers to ignore their future gestures and utterances if they lack decisional capacity when those utterances are made.^60^

The NJP directive squarely addresses this important issue by asking the individual what they want their medical and/or care team to do if they make an utterance or gesture requesting food or drink after food and drink are withheld.^61^ Surely, the person most appropriately suited to address this nuanced question is the same person who has affirmatively chosen to create an advance directive that directs the future withholding of food and drink so that they do not live into severe dementia.

## Ethical Justification of VSED Directives

The right to VSED is a moral, not just a legal, right. Ethically, not just legally, it would be wrong for others to prevent people from exercising it. Practically speaking, the right to VSED is implied by the widely accepted right to refuse medical treatment, for medically delivered nutrition and hydration is the only way, ultimately, to force-feed a patient who refuses to eat or drink, and one has the right to refuse that.^62^

The accepted ethical justification for a person’s right to VSED is based on bodily integrity.

We each have the right to refuse life-sustaining treatment, even if we are not terminally ill, because our body is ours, and we alone have the right to make that decision.^63^

Pair this right to bodily integrity with the moral right that we each can determine what is done to us after we lose decision-making capacity, and one has the essential ethical justification of VSED by advance directive. You do not lose your right to refuse lifesaving treatment when you lose capacity. Rather, others must exercise that right for you (typically your healthcare agent or other legally designated decision-maker). Similarly, you do not lose your right to stop eating and drinking. The reason is not that manually assisted oral feeding and drinking is “medical treatment” governed by the right to refuse treatment, but rather that refusing to eat and drink is your moral and legal right notwithstanding whether providing food and drink is “medical treatment.”

### “Then-Self vs. Now-Self” Problem

A well-known objection to the moral authority of advance directives is the so-called “then-self vs. now-self” problem.^64^ In the dementia directive context, it occurs when, in accordance with a clear and applicable directive, oral food and drink are withheld, but the person behaviorally expresses a desire to drink or be fed. Should we still honor the directive, or should we provide enough food and liquid to satisfy the desire? Note that this problem can be a challenge not only for directives for VSED but also for traditional directives to refuse treatment. It must be solved, or we will have greatly weakened the authority of all advance directives.

And it can be solved. We can see this by breaking the problem into four layers, all of which can be addressed persuasively.

(1) Revocation. Some might claim that in expressing a desire to eat or drink, the person has revoked the directive. They have not. To revoke a directive, one must understand what one is revoking, and that what one is doing is revoking it. The person in advanced dementia is not capable of either.

(2) Change of Mind. In this more colloquial version, though short of revocation, the person has had a “change of mind.” In changing her mind, in the normal sense of that notion, the patient here would need to take a different attitude toward surviving into advanced dementia, or have a different belief about it. In expressing an immediate desire for food or drink, the person has done neither. As one commentator poignantly puts it, “at the time you would most likely ‘change your mind’ [about your directive], you don’t have enough mind left to change.”^65^

(3) Different Persons. Suppose Charles has progressed to advanced dementia (FAST stage 7).^66^ Someone then says, “The earlier Charles isn’t here anymore. He’s a different person now.” After all, virtually none of Charles’ personality remains. If psychological similarity and connectedness through time is required for identity of person, the new Charles may indeed be a different person, and if so, why should the wishes of the other, past person control what now happens to Charles?

The problems with this argument are multiple. Psychological similarity cannot be the criterion for personal identity. If it were, we would think Charles at 60 (or even 30) literally a different person than Charles at 3, but we don’t. And when Charles died, we would hold a memorial for two persons, the old Charles and the new. (Or two memorials, one for the old Charles as the new one emerged, and one when new Charles dies.) But we don’t hold multiple memorials. We hold one, for the one person whose time between birth and death constitutes Charles’ life. It may contain huge variety and change. But it is one single life.

This is belied by our very speech. We speak of the Charles we visit in the nursing home as still the Uncle Charles we have known through the decades. Admittedly, he’s very different now, but he’s still our Uncle Charles. That’s why we visit him. Deeply within everyday thought and language, then, we reject the view that the person in advanced dementia is a different person than the one who wrote the directive. In fact, these presumptions about identity constitute the basic intuition behind the very notion of advanced directives. Advance directives (ADs) represent a claim to control by people over *their* later lives. The continuing identity of persons is why directives have any plausibility at all.

(4) Desires, Choices, Values. In one prominent view, notable in the work of Ronald Dworkin, the patient’s expressed desire forms a current “experiential” interest in being fed, distinguished in kind from the “critical” interests formed in the more reflective choices and values expressed in an AD.^67^ We do not have to accept the full distinction and Dworkin’s view that critical interests are always more important than experiential interests in respecting patient autonomy. We still appreciate that the choices and values formed with considerably greater thought than a momentary desire to eat or drink are more important in respecting the patient as person. The apparent desires to eat and drink when one is in advanced dementia do not constitute one of the person’s values, or even a choice. The choices and values expressed in the directive constitute a more significant and longstanding aspect of the person.

Two related points are fundamental in resolving the then-self/now-self problem. Why do we have a requirement of informed consent and the right to VSED to begin with? These rest on self-ownership: for each of us, our bodies are ours, and our lives, not just moments in our lives, are ours. For those who have ADs to withhold oral food and drink in advanced dementia, whether their lives end with years of such dementia or not is a truly significant, not a relatively incidental, aspect of who they are. In terms of their values, they are harmed if they must live into those years.

Similarly fundamental in solving the then-self/now-self problem is understanding that even now, the patient before us who poses the problem is the same person who wrote the directive. Treating the patient as she is now, therefore, must include treating her as a person who has this directive.^68^ The moral weight of advance directives is thus preserved, even when they conflict with some of a patient’s current desires.

### Minimal Comfort Feeding (MCF)

An important option related to this is provided in the new directive as well: “minimal comfort feeding” (MCF).^69^ In “comfort feeding only” (CFO), an approach frequently used for those in advanced dementia, no more food and drink than is comfortable for the patient is provided. In MCF, only what is necessary to avoid discomfort due to hunger or (more commonly) thirst is provided.^70^ Many people who receive CFO live for many months, even years. In contrast, MCF results in survival for only weeks or a few months. MCF is therefore a logical “fallback” when clinicians or facilities fail to implement a VSED directive.

Even in its own right, not just as an option when the directive’s implementation is blocked, MCF has major attractions. Clinically, those who faithfully, assiduously feed severely demented patients for months and years on end may find that abiding by an advance directive for full withholding is a violation of their moral obligation to compassionately care for the patient. MCF, well explained, allows them to reconcile restraints on feeding with their moral beliefs, since enough food and drink to maintain comfort is provided. Morally, MCF’s attraction may be even greater. It literally avoids the then-self/now-self problem. If enough food and drink were provided to avoid discomfort and satisfy the patient’s desire, as is provided in MCF, there is no conflict with the current patient’s needs.

That said, with the moral weight of advance directives thus preserved, the situation where the person desires to eat and drink despite their directive must be recognized as a morally significant conflict. The directive itself should address the conflict. Don’t dodge the problem. Don’t claim it’s not a serious one. Don’t allege that it can be definitively solved. Confront it explicitly. Accordingly, the new NJP directive asks directly: what should be done if one later expresses a desire for food and drink when food and drink are being withheld?^71^ If the person chooses either “continue to withhold all food and drink” or “provide MCF,” it is clear what they would have wanted.

## Limitations and Next Steps

The NJP directive helps assure value congruent care. But it may not be an easy path to follow.

Caregivers may find it emotionally difficult to refuse food and water to the patient. They may suffer moral distress by ignoring the patient’s apparent discomfort. Family caregivers may even hand feed the patient or direct that professional caregivers do so. Moreover, providers typically look for ethical assurance to their professional societies’ endorsements or guidelines about controversial measures like limiting food and drink.^72^ In short, even with the NJP directive and a carefully appointed surrogate, one cannot be 100% confident of successful implementation.

This is especially true in long-term care facilities where policies to operationalize the withholding of food and drink by advance directive have not been developed.^73^ So more must be done to protect and promote patient autonomy. Here are the first five steps. First, institutions must develop policies and procedures for VSED by advance directive. Second, institutions must be disabused of the myth that state and federal regulations prohibit VSED. In fact, rights to adequate nutrition and hydration can be waived. And they are waived by patients with VSED directives.^74^ Third, more training on VSED and advance directives is needed for implementation to come close to being assured. Indeed, more training on advance directives and dementia is needed more broadly.^75^

Fourth, palliative support measures, including proportionate palliative sedation, must be understood as part of good care for patients who have chosen to forego eating and drinking.^76^ Fifth, caregivers, especially those who devotedly, day in and day out, manually feed patients in advanced dementia, should understand that in continuing assisted feeding, they are failing to honor the wishes of the patient they are caring for; with such understanding, they may come to moderate their reluctance to withhold assisted feeding.

These are significant challenges and limitations. Nonetheless, the NJP directive is an important step in providing people with increasingly effective means to avoid living into advanced dementia.

## Conclusion

A thorough and enforceable dementia directive can provide a preferred alternative to preemptive VSED. It will help people with progressive dementia achieve their aim of not living into dementia’s advanced stages without the need to sacrifice months or even years of good living for fear that their advance directive will not later be honored. The NJP dementia directive addresses issues that plague other published dementia directives upon implementation. Of course, as with any directive, implementation will be assisted by appointing a healthcare agent (and alternate agent) in an advance directive or Durable Power of Attorney for Healthcare.

## Supporting information

Pope et al. supplementary material 1 Pope et al. supplementary material

Pope et al. supplementary material 2 Pope et al. supplementary material

Pope et al. supplementary material 3 Pope et al. supplementary material

## Supplementary Material

The supplementary material for this article can be found at http://doi.org/10.1017/jme.2025.10176.

## Note

Robb Miller is Co-Chair of the National Voluntary Stopping Eating and Drinking Committee and has received honoraria for the provision of presentations and one-on-one consultations related to general advance planning, including advance directives, dementia directives, voluntarily stopping eating and drinking, and Portable Orders for Life Saving Treatment from Worry Free Wednesday. Paul T. Menzel is on the advisory board of The Completed Life Initiative. Thaddeus Mason Pope receives honoraria for presentations on VSED to professional societies and healthcare systems. And with Paul T. Menzel he receives royalties for their Oxford University Press book on VSED. The other authors have nothing to disclose.
