# Supplementary material for: New VSED Advance Directive: Improved Documentation to Avoid Late-Stage Dementia
Source: J Law Med Ethics. 2025 Winter;53(4):491–9. doi: 10.1017/jme.2025.10176 (PMC12914199; doi:10.1017/jme.2025.10176)
Supplement: Pope et al. supplementary material 1 — Pope et al. supplementary material [file S1073110525101769sup001.pdf]

Advance Directive for Voluntary Stopping of Eating and Drinking (VSED)

Author
Northwest Justice Project

Last Review Date
November 26, 2024

A VSED Directive expresses your end-of-life wishes if dementia or other progressive illnesses cause you to lose the ability to make your own health care decisions. This VSED Directive can be used anywhere in the United States. (Form and instructions)

1. Fast facts

Form attached:
Advance Directive for Voluntary Stopping of Eating and Drinking (VSED Directive) (NJP Planning 515)

What is VSED?

VSED stands for Voluntary Stopping of Eating and Drinking. It means you voluntarily refuse to eat food or drink liquids, with the understanding that it will accelerate the process of dying and reduce suffering. People with a serious, irreversible, intolerable condition, for instance, might choose VSED.

What is a VSED Directive?

A VSED Directive is a document that expresses your end-of-life wishes about receiving food or drink from care providers.

Why would I choose VSED instead of (or in addition to) medical aid in dying?

Some progressive illnesses, like dementia, make you lose your ability to make decisions about your health care, including choosing medical aid in dying. VSED does not require a specific diagnosis or physician order, so it might be the only option for some people wishing to accelerate the process of dying.

What if I already have a health care directive?

Your VSED Directive does not replace your other advance directives, such as health care or mental health advance directives. It also doesn't replace other documents (like a POLST or MOLST form) that decline emergency medical treatment.

Is a VSED Directive legal?

Yes. A VSED Directive is a legal advance directive. Medical providers are usually legally required to follow your advance directives. The best way to make sure your wishes will be honored is to talk with your medical team, your

care facility, your caregivers, and your family members about your VSED Directive.

2. Step-by-step

1. **Fill out your VSED Directive form**. You can fill it out online (https://walawhelp.gavel.io/start/playground2/VSED%20Directive), or download and follow the instructions in the next section.

2. **Sign in front of a notary and/or two witnesses.**

3. **Where to keep your directive**: Unlike most legal documents, copies of directives are just as valid as the original. Keep the original signed documents in a secure but accessible place. Your directives are useless if they can't be found.

   Tip! Ask your health care decision-maker to keep a digital copy of your signed directives on their smartphone for easy access in the event of a medical emergency. You may also ask them to keep a copy in the glove box of their car, if they have one, and in their suitcase.

4. **Who should have a copy?** Make copies of your directive and share it with people involved in your care. This may include your health care decision-maker, doctors, lawyer, family, close friends, clergy, and any facility that might be involved in your care.

If you can, attach a copy of your Power of Attorney or other legal document naming a health care decision-maker to your directive. Make sure there is a copy of your directive on file at your local hospital and your long-term care or memory care facility. If your state has an advance directive registry online, post your directive there.

5. **Tell important people about your wishes**. It is extremely important for you to tell your health care decision-maker and anyone else who may be involved in making decisions related to your directive what your wishes are and why you are making those decisions. Repeat these conversations periodically, especially if your medical condition changes.

> Although not required to complete this directive, we highly recommended you name a health care decision-maker whom you trust to honor your wishes.

Make clear to your close friends and relatives that your health care decision-maker will have final authority to act on your behalf. Emphasize that you don't want them to disregard or undermine your wishes because they think your quality of life is acceptable or because you appear to be happy or comfortable. If your health care decision-maker is not supportive or disagrees with the decisions you make in your directive, you should appoint someone else as your health care decision-maker.

6. **Will the doctor or long-term care facility honor your VSED Directive?** When you give your directive to your physicians, ask if they will honor it.

If not, find a physician who will. If you need care in a nursing home, memory care facility or other long-term care facility, choose one that agrees to honor your VSED Directive. If you can't, instruct your health care decision-maker to try to find a facility that will honor your VSED Directive. If your current facility changes its policy on VSED, you can also instruct your health care decision-maker to move you to another facility or home.

7. **If you are admitted to a health care or long-term care facility or enrolled in a home-based health care or hospice program**: Give admissions staff a copy of your completed VSED Directive and any other directives. Also tell your health care decision-maker to give admissions staff a copy if you are admitted *after* you lose capacity.

8. **Make sure your POLST, MOLST or other form allowing you to decline treatment by emergency medical providers doesn't contradict your VSED Directive**: Many states offer a form that allows you to express your wishes for emergency medical treatment, such as CPR. Unlike advance directives, these forms must be signed by a doctor or other qualified medical provider. Many of these forms include a section about nutrition and hydration that may also include language such as "Food and liquids to be offered by mouth if feasible…" That language may contradict your VSED Directive. If language like this appears in your POLST, MOLST or other emergency medical treatment form, you should cross it out and initial the change.

9. **Keep your directives updated**. Be sure to occasionally review your VSED and other directives to be sure they reflect your current preferences and values. Initial and date it whenever you review it.

   Tip! Create a calendar reminder to review your directives every 1-2 years.

10. **Canceling (revoking) your directive**: If you revoke a directive, make sure you notify your health care decision-maker, family, and doctors. If possible, retrieve and destroy copies of your revoked directive, or tell those who have revoked copies to destroy them. Keep one copy of your revoked advance directive in your records with the word “REVOKED” written across the front. This could help if someone needs to rely on a new directive. The most recently dated directive will be honored over any older directive.

3. Completing the form

> Try using WA Forms Online (https://walawhelp.gavel.io/start/playground2/VSED%20Directive)! It's simple: we ask you questions and use your answers to complete your form.

If you **don’t** want to fill it out online, follow these instructions:

Put your name and birth date in the blanks.

Directive section 1. Voluntary stopping of eating and drinking (VSED)

Section 1 describes what you are asking your caregivers and health care providers to do.

Directive section 2. Conditions for starting VSED

First, decide which conditions will trigger the start of VSED, when you want your caregivers to stop giving you food and drink.

Then initial by the specific triggering conditions you want to select. You can add other conditions by initialing the last option and filling in the blank. For example, you might add the following condition: “I do not remember that a particular person came earlier today, or yesterday, or any time during the past week.”

The conditions listed typically occur in advanced stages of dementia, but they might characterize other illnesses as well. They are described in ways that ordinary people understand them; they are not “clinical” or medical descriptions. If you want to include more clinical or medical descriptions, you can use one or more of the stages found in the Global Deterioration Scale (GDS) or the Functional Assessment Staging Tool (FAST).

Directive section 3. If my decision-maker thinks my quality of life is still good when it is time to start VSE

You can decide if you want your decision-maker to follow your directive no matter what, or if you want them to be able to ignore it if they believe your quality of life is good enough. “Quality of life” means different things to different people. What your decision-maker might consider acceptable may not be acceptable to you.

Initial the option you want.

Directive section 4. Palliative care – relief from pain and discomfort

You will need palliative care to manage the discomfort of going without food and drink. This includes medication and other ways to provide comfort.

> Palliative care may include proper mouth care, repositioning for physical comfort, massage, acupuncture, and sensory and mental stimulation including visits with loved ones, music, movies, and reminiscing over photo collections. VSED Resources Northwest has more ideas (https://vsedresources.com/education-outreach/vsed-comfort-measures).

In addition to standard palliative care, you may request more intensive palliative care if needed. This could include palliative sedation to *reduce consciousness*, and even palliative sedation to *unconsciousness*.

Initial the option you want.

Directive section 5. If I express the desire to eat or drink

If your memory fails, you may not remember your VSED Directive and may express a desire to eat or drink after your caregivers have stopped giving you food and drink.

Initial the option you want.

Directive section 6. Medical facilities and providers that will not honor this directive

VSED can be controversial. Some laws and policies allow health care providers to refuse to comply with requests that go against their religious or moral values, even when the requested health care is legal and generally accepted.

You can **also** say if you only want to get care from a provider that will honor your VSED Directive, even if this means transferring you to another facility or provider. Initial the "After I am admitted or receiving care…" paragraph if you agree.

Directive section 7. Dispute resolution

If there is any disagreement about what your directive means, this section explains how to resolve it. You are giving your decision-maker the authority to make a final decision on the meaning of your VSED Directive.

Directive section 8. Health care decision-maker

The single most important decision you can make to ensure your directive will be honored is to name a decision-maker in a legal document (like a power of attorney) who will advocate for the choices you made. Depending on where you live, this person may be called a health care agent, proxy, representative, or surrogate.

You must use the correct legal document in your state to give your decision-maker legal authority. In Washington State, the correct document is a Durable Power of Attorney that gives a trusted person ("agent") the power to make health care decisions.

Initial if you have already named a health care decision-maker in a legal document and write in what the legal document is called. If possible, attach a copy of that legal document to your VSED Directive.

Make sure your health care decision-maker is aware that you are making this VSED Directive. Give them a copy and discuss it with them to make sure they understand and are willing to honor it. A health care decision-maker who is comfortable making other end-of-life decisions for you **may not** be willing to uphold your decision to stop eating and drinking. In that case, look for a new health care decision-maker who will be.

Warning! If you **don't** name a health care decision-maker in a legal document, someone else will be given the legal authority to make decisions on your behalf. That person may have less power to make decisions. Also, it could be someone you don't want to make decisions for you.

Directive section 9. Other advance planning

Your VSED Directive does not replace your other advance directives such as a living will, health care directive, or mental health advance directive. It also does not replace any documents about emergency medical treatment such as a POLST or MOLST form. (Different states call these forms by different names.)

List your other advance planning documents in your VSED Directive so your caregivers and providers are aware of them.

Directive section 10. Liability waiver

Your decision-maker, medical providers, caregivers, family and loved ones may be concerned about their legal responsibility for implementing this directive. In this section you release them from liability for following your

instructions. You also authorize a lawsuit against anyone who provides food and water against your instructions.

Directive section 11. Capacity

This section explains that you understand and mean what you say in your directive.

Signature

Don't sign until you can do it in front of witnesses and/or a notary.

Notarization and Witnessing

It is best to sign in front of a notary **and** witnesses. This gives you the greatest assurance that it will be honored as a legally valid directive. That said, if that is too difficult, most states will honor it if it is either witnessed **or** notarized. Your state's rules for notarizing and witnessing other health care advanced directives apply to this document.

WashingtonLawHelp.org gives general information. It is not legal advice.
Find organizations that provide free legal help on our Get legal help page.

Advance Directive for Voluntary Stopping of Eating and Drinking (VSED Directive)

My name is ___________________________________. My date of birth is ________________.

As an adult with decision-making capacity, I have the right to direct my treatment and care, even if those choices lead to an earlier death. This includes the right to refuse medical treatment and the right to refuse oral food and drink. I have thought carefully about the circumstances in which I would want to stop prolonging my life with eating and drinking.

This directive instructs my health care agent or other legal decision-maker (“decision-maker”) and all caregivers how to act on my behalf to ensure that my wishes for stopping eating and drinking are carried out.

1. Voluntary stopping of eating and drinking (VSED)

When I meet the conditions I have selected in section 2 (below) and can no longer feed myself:

- Do not help me with eating and drinking (by spoon-feeding, for example).
- Do not verbally or physically encourage or persuade me to eat or drink.
- Do not put food or liquids in my mouth.

2. Conditions for starting VSED

I want to start VSED when I have a serious and irreversible illness or chronic condition that will not significantly improve (even if it is not terminal), and when I meet (*initial one*)

__ **at least one** of the conditions I select below.

__ **all** of the conditions I select below

(*initial all that apply*):

__ I cannot communicate with others beyond a few words, eye movements, etc.

__ I do not recognize close family and friends.

__ I am indifferent to being fed, no longer want to eat or drink, and show no signs of enjoying eating and drinking.

__ I do not open my mouth to receive food and drink, or I turn my head away when offered food or drink.

__ I usually refuse food or drink

__ I frequently inhale or choke on food or drink.

__ The following additional conditions or situations:

____

© Northwest Justice Project, National VSED Advance Directive Committee

3. **If my decision-maker thinks my quality of life is still good when it is time to start VSED**

If my decision-maker thinks my quality of life is still good enough and I seem comfortable or happy, my decision-maker (*initial one*):

__ **must follow this directive and start VSED.** I have given a lot of thought to this decision and insist that my wishes be followed.

__ may choose **not** to follow this directive. **I understand this means some or all of my choices may not be honored.**

4. **Palliative care – relief from pain and discomfort**

If food and drink are being withheld, I want palliative care to manage any pain or discomfort from my illness and from not eating and drinking (relief from dehydration, for instance).

I want palliative sedation if necessary to manage pain and discomfort (*initial one*):

__ **even if** it makes me unconscious.

__ **but not** to the point of unconsciousness.

5. **If I express the desire to eat or drink**

If eating and drinking has stopped, but I repeatedly show by words or gestures that I want to eat or drink, I want my caregivers to reassess my palliative care and (*initial one*):

__ continue to withhold all help with eating and drinking.

__ give me only enough food and drink to avoid discomfort, even if it's not nutritionally adequate (also known as 'minimal comfort feeding'). **I understand this approach will likely prolong my dying process.**

6. **Medical facilities and providers that will not honor this directive**

Before I receive care from a medical facility or provider (including my physician or residential hospice, or long-term care facility), I want the facility or provider to confirm it will follow the instructions in this directive. If the medical facility or provider will not follow the instructions in this directive due to moral, ethical, or other reasons, my decision-maker should make all reasonable efforts to make sure I get care from a facility or provider that will.

(*Initial if selected*)

__ **After** I am admitted or receiving care, if a facility or provider will not honor the instructions in this directive, my decision-maker should make all reasonable efforts to make sure I get care from another facility or provider that will. I understand this means I may be transferred to another medical facility or living situation that might cost more or be less convenient.

If a medical facility or provider will **not** follow this directive due to legal or institutional barriers, I want to be given only enough food and drink to avoid discomfort even if not nutritionally adequate (also known as 'minimal comfort feeding').

© Northwest Justice Project, National VSED Advance Directive Committee

7. **Dispute resolution**

My decision-maker will resolve any disagreement about the instructions in this directive and/or whether the conditions I have chosen have been met.

If no decision-maker is available, then I want my medical providers to make these decisions if that is legally allowed.

If any part of this directive is determined to be legally invalid, all other parts should be honored.

8. **Health care decision-maker**

__ I have named a health care decision-maker in the following legal document (*initial one and attach a copy, if possible*):

__ Power of Attorney for Health Care

__ Other document (*name*): ____

__ I have **not** yet named a health care decision-maker.

9. **Other advance planning**

I intend this directive to supplement any existing documents about my end-of-life care. This directive does not revoke any existing documents except with respect to receiving food and liquid by mouth, in which case this directive shall govern.

I have the following other documents about advance planning or end-of-life wishes:

Name: ___________________________________

Name: ___________________________________

Name: ___________________________________

Name: ___________________________________

10. **Liability Waiver**

I voluntarily assume any and all risk associated with my choice to use VSED as an end-of-life option. I release all persons, including my health care decision-makers, medical providers, caregivers (including but not limited to my physicians, nurses, care facilities, doulas, or personal care providers, etc.), and family members and other loved ones from any and all liability that could result from any and all actions they may take in good faith reliance on my wishes as described in this directive. This includes my express release of civil liability and my strongly held wish that they **not** be subject to any criminal or disciplinary sanctions.

Further, I direct my estate to hold harmless and indemnify my health care decision-makers, medical providers, caregivers, family members, and other loved ones for acts done according to this advance directive in good faith.

Finally, I wish to make it clear that I am making this directive of my own free will and am doing so intentionally to ensure that my medical care is consistent with my stated wishes. As a result, I regard any action taken to undermine my wishes as described in

© Northwest Justice Project, National VSED Advance Directive Committee

this directive as medical battery and authorize my surrogate and/or my estate to pursue such a claim on my behalf.

11. Capacity

I am making this VSED Directive because if I cannot make decisions for myself, I want my decision-makers, medical and long-term care providers, caregivers, family, and other loved ones to honor every part of this directive.

I am of sound mind. I am voluntarily signing this VSED Directive and understand what it means. I make this advance directive of my own free will, and I have the mental and emotional capacity to do so.

I understand that honoring this directive might cause me to die sooner than if I received help with eating and drinking.

[Sign only in the presence of a notary or qualified witnesses]

______________________________ ▶______________________________

Date My Signature

____

Print name

➢ Notarization and Witnessing

While it is best to have this document both notarized and witnessed, in most places this document is legally binding with either one or the other.

Notarization

State of ______________________

County of _____________________

Signed or attested before me on (*date*): __________________

by (*name*):____.

▶______________________________

Signature of Notary

Notary Public for the State of Washington.

My commission expires ___________________.

© Northwest Justice Project, National VSED Advance Directive Committee

Statement of Witnesses

I, the witness, declare that the person who signed or acknowledged this VSED Directive:

- Is personally known to me
- Signed or acknowledged this VSED Directive in my presence
- Appears to be of sound mind and under no duress, fraud, or undue influence

I also declare that I am over 18 years of age (19 in Alabama) and that I am:

- **Not** the person's health care agent, decision-maker, or alternate decision-maker
- **Not** the person's health care provider, including an owner or operator of their long-term care, residential, or community care facility
- **Not** an employee of the person's health care provider
- **Not** financially responsible for the person's health care
- **Not** an employee of a life or health insurance provider for the person
- **Not** related to the person by blood, marriage, or adoption
- **Not** a beneficiary of any legal instrument, account, or benefit plan of the person
- **Not** a creditor of the person or entitled to any part of their estate under a will or codicil, by operation of law

(*Some states may have different rules about who may be a witness. Unless you know your state's rules, please follow the above.*)

Witness 1

▶ ________________________________
Signature

Print name: ________________________

Address: __________________________

____

Phone: __________________________

Witness 2

▶ ________________________________
Signature

Print name: ________________________

Address: __________________________

____

Phone: __________________________

© Northwest Justice Project, National VSED Advance Directive Committee
