# Supplementary material for: New VSED Advance Directive: Improved Documentation to Avoid Late-Stage Dementia
Source: J Law Med Ethics. 2025 Winter;53(4):491–9. doi: 10.1017/jme.2025.10176 (PMC12914199; doi:10.1017/jme.2025.10176)
Supplement: Pope et al. supplementary material 2 — Pope et al. supplementary material [file S1073110525101769sup002.docx]

Appendix 1. **Advance Directive for Voluntary Stopping Eating & Drinking (VSED)**

Northwest Justice Project

This directive instructs my health care agent or other legal decision-maker and all caregivers how to act on my behalf to ensure that my wishes for stopping eating and drinking are carried out.

1. **Voluntary stopping of eating and drinking (VSED)**

When I meet the conditions selected in section 2 (below) and can no longer feed myself:

- - Do not help me with eating and drinking (by spoon-feeding, for example).
  - Do not verbally or physically encourage or persuade me to eat or drink.
  - Do not put food or liquids in my mouth.

1. **Starting VSED.** I want to start VSED when I have a serious and irreversible illness or chronic condition that will not significantly improve (even if not terminal), **and** when I meet (*initial one*)

**___ at least one** of the conditions I select below.

**___ all** of the conditions I select below (*initial all that apply*):

I cannot communicate with others beyond a few words, eye movements, etc.

I do not recognize close family and friends.

I am indifferent to being fed, no longer want to eat or drink, and show no signs of enjoying eating and drinking.

I do not open my mouth to receive food and drink, or I turn my head away when offered food or drink.

I usually refuse food or drink.

I frequently inhale or choke on food or drink.

The following additional conditions or situations:

_________________________________________________________

1. **If my decision-maker thinks my quality of life is still good when I have met the above designated conditions** and I seem comfortable or happy, my decision-maker (*initial one*):

**must follow this directive and start VSED.** I have given a lot of thought to this decision and insist that my wishes be followed.

may choose **not** to follow this directive. **I understand this means some or all of my choices may not be honored.**

1. **Palliative care to relieve pain and discomfort**

If food and drink are being withheld, I want palliative care to manage any pain or discomfort from my illness and from not eating and drinking (relief from dehydration, for instance).

I want palliative sedation if necessary to manage pain and discomfort (*initial one*):

**___ even if** it makes me unconscious.

**___ but not** to the point of unconsciousness.

1. **If I express the desire to eat or drink**

If eating and drinking has stopped, but I repeatedly show by words or gestures that I want to eat or drink, I want my caregivers to reassess my palliative care and then (*initial one*):

___ continue to withhold all help with eating and drinking.

___ give me only enough food and drink to avoid discomfort, even if it's not nutritionally adequate (also known as 'minimal comfort feeding'). **I understand this approach will likely prolong my dying process.**

1. **Medical facilities and providers that will not honor this directive**

**Before** I receive care from a medical facility or provider (including my physician, residential hospice, or long-term care facility), I want the facility or provider to confirm it will follow the instructions in this directive. If the facility or provider will not follow the instructions in this directive due to moral, ethical, or other reasons, my decision- maker should make all reasonable efforts to make sure I get care from a facility or provider that will.

Furthermore (*initial if selected*):

___ If, **after** I am admitted or receiving care, a facility or provider will not honor the instructions in this directive, my decision-maker should make all reasonable efforts to make sure I get care from another facility or provider that will. I understand this means I may be transferred to another medical facility or living situation that might cost more or be less convenient.

If a medical facility or provider will **not** follow this directive due to legal or institutional barriers, I want to be given only enough food and drink to avoid discomfort even if that is not nutritionally adequate.

1. **Dispute resolution**

My decision-maker will resolve any disagreement about the instructions in this directive and/or whether the conditions I have chosen have been met.

If no decision-maker is available, then I want my medical providers to make these decisions if that is legally allowed.

If any part of this directive is determined to be legally invalid, all other parts should be honored.

1. [Other standard sections follow on health care agent, other advance directives, liability waiver, capacity, witnesses and/or notary, etc.]
